# Transperitoneal Laparoscopic and Robotic Partial Nephrectomy for Renal Cancer in Patients with Previous Abdominal Surgery: a Single Centre Experience

**DOI:** 10.1007/s12262-023-03743-x

**Published:** 2023-04-27

**Authors:** Lukas Gadus, Frantisek Chmelik, Marketa Matejkova, Jiri Heracek

**Affiliations:** 1 Department of Urology, Military University Hospital, 16902 Prague, Czech Republic; 2 First Faculty of Medicine, Charles University, 12108 Prague, Czech Republic

**Keywords:** Renal cancer, Laparoscopic partial nephrectomy, Robotic partial nephrectomy, Previous abdominal surgery, Indocyanine green

## Abstract

Patients with previous abdominal surgery are at an increased risk of peritoneal adhesions, which may complicate transperitoneal surgery. The objective of this article is to report single centre experience with transperitoneal laparoscopic and robotic partial nephrectomy for renal cancer in patients with previous abdominal surgery. We evaluated data from 128 patients who underwent laparoscopic or robotic partial nephrectomy from January 2010 to May 2020. Patients were divided into three groups according to the localization of main previous surgery: in the upper contralateral abdominal quadrant, in the upper ipsilateral abdominal quadrant or in the middle line, in lower abdominal quadrants. Each group was divided into two subgroups (laparoscopic/robotic partial nephrectomy). We separately analysed data of indocyanine green-enhanced robotic partial nephrectomy. Our study did not find significant difference in the rate of intraoperative or postoperative complications between any of the groups. The type of partial nephrectomy (robotic or laparoscopic) affected the surgery time, blood loss, and length of stay in hospital, but did not significantly influence the frequency of complications. Partial nephrectomy in group of patients with prior renal surgery led to a higher rate of intraoperative low-grade complications. We did not observe more favourable results for indocyanine green-enhanced robotic partial nephrectomy. The location of previous abdominal surgery does not influence the rate of intraoperative or postoperative complications. The type of partial nephrectomy (robotic or laparoscopic) does not affect the frequency of complications.

## Introduction

Patients with a history of previous abdominal surgery are at possible risk of perioperative complications during subsequent surgical procedures [1, 2]. Previous groups have reported the effects of previous abdominal surgery on transperitoneal laparoscopic or robotic urologic procedures including surgical treatment for renal cancer with variable results [1–3]. The aim of our work is to provide an extensive and complex view of outcomes and complications for both laparoscopic and robotic transperitoneal partial nephrectomies, and to identify which subgroup has the highest possibility for developing intraoperative or postoperative complications. We evaluated perioperative statistics and postoperative results of partial nephrectomies in patients with a history of previous renal surgery and in patients with a history of previous non-renal surgery separately.

Modern digital visualisation technologies have facilitated the usage of perioperative navigation and imaging in order to achieve more favorable functional results during surgery. One common method of perioperative imaging is near-infrared fluorescence with indocyanine green (ICG), based on the standard principle of fluorescence, as described well in the literature [4]. ICG permits the surgeon to perform perioperative angiography with precise vascularity visualisation, imaging of anatomical features of tissues, and lymphography with ICG-navigated sentinel lymph node mapping [5]. Our centre is focused on the principle of ICG-angiography during robotic partial nephrectomy, which enables selective clamping of the segmental renal artery or superselective clamping of the tumour-supplying artery. Thus, we separately introduce and evaluate outcomes and complications in patients with previous abdominal surgery who underwent robotic partial nephrectomy enhanced by ICG usage.

## Patients and Methods

### Patients

We retrospectively evaluated data of 219 patients who underwent laparoscopic or robotic partial nephrectomy in our centre from January 2010 to May 2020. We have chosen for the end of data evaluation period the beginning of COVID-19 disease in our country, which significantly affected surgical performance in our department. We identified 128 patients who had at least one previous abdominal surgery in their personal history and who underwent laparoscopic or robotic partial nephrectomy for a renal tumour. A total of 28 patients underwent robotic surgery with the use of ICG (this fluorescent dye has been used in our centre since April 2015). One patient underwent a robotic partial nephrectomy using a retroperitoneal approach, and was not included in the study. The data are analysed with delay due to COVID-19 pandemic and restrictions.

### Data and Statistical Analysis

The patients were divided into three groups according to the localization and type of previous surgery and its proximity to the site of the partial nephrectomy: (A) patients with a main previous surgery in the upper contralateral abdominal quadrant, (B) patients with a main surgery in the upper ipsilateral abdominal quadrant or in the middle line, (C) patients with a main previous surgery in lower abdominal quadrants. Each group was divided into two subgroups according to the type of partial nephrectomy (laparoscopic/robotic), and intraoperative and postoperative outcomes were analysed separately. A total of 57% patients had undergone one previous abdominal surgery, while 43% had undergone two or more previous surgeries. In cases with multiple surgeries in the personal history, we considered the prior surgery located at the shortest distance to the field of planned partial nephrectomy as definitive for dividing the patients into the respective groups. We separately evaluated outcomes and intraoperative and postoperative complications of ICG-enhanced partial nephrectomies. Intraoperative and postoperative outcomes of patients with a history of previous renal surgery and patients with a history of previous non-renal surgery were evaluated separately as well. In all groups, we recorded mean age, body mass index (BMI), Charlson comorbidity index (CCI) and tumour diameter. We evaluated mean surgery time, warm ischemia time, blood loss, length of hospital stay, positive surgical margins, intraoperative and postoperative complications, preoperative and postoperative creatinine value. Intraoperative and postoperative complications were evaluated according to Clavien-Dindo (CD) classification of surgical complications [6]. Variables are presented as mean ± standard deviation and median (range). The mean values were compared using the Student’s *t*-test. All test were performed in STATISTICA, version 12, Statsoft Inc, Tulsa, CA. The level of significance was set at 0.05.

### Surgical Technique

**Fig. 1:**
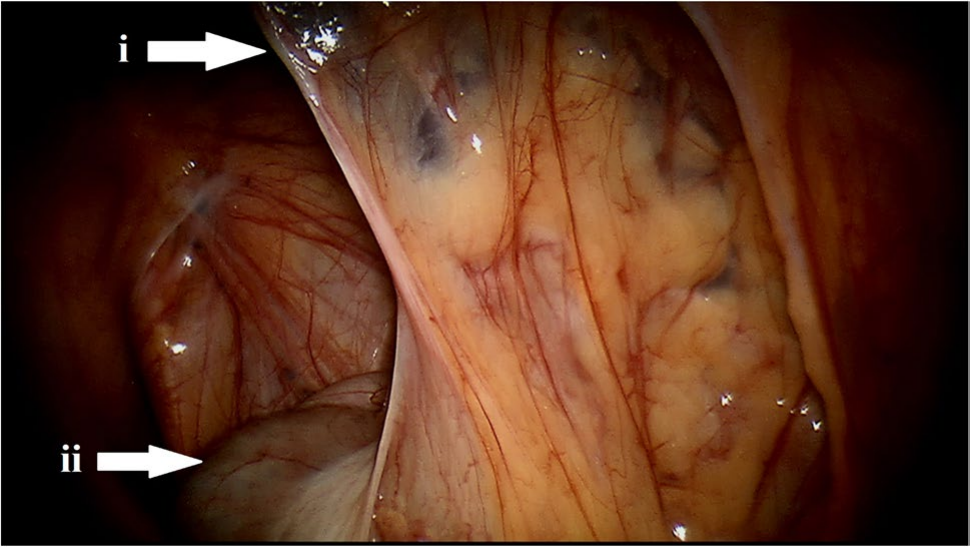
Surgeon’s view of peritoneal adhesions (i) of the colon (ii) after cholecystectomy during adhesiolysis before the robotic partial nephrectomy

Laparoscopic partial nephrectomy was started by placing the patient on the contralateral side position. A pneumoperitoneum was created via a Veress needle, inserted into non-scarred skin, and the abdominal cavity was insufflated to 13 mmHg. Then, a camera port (12 mm) was inserted through the incision. If the pneumoperitoneum could not be created by the Veress needle, the Hasson technique was used for insertion of the camera port [7]. Two ports for laparoscopic instruments (5 mm, 12 mm) were placed in the medioclavicular line. Adhesiolysis of recognised adhesions (Fig. 1) was performed with laparoscopic scissors. Complete exophytic renal tumours were dissected without hilar arterial clamping, endophytic renal tumours were dissected with non-selective, selective or superselective clamping according to the current renal vascular anatomy. Renal calices were repaired if damaged, a running suture ensured by hem-o-lock clips over the resection bed was done to stop bleeding and to attach the margins of resection. A bag was used for tumour extraction, pneumoperitoneum was evacuated, fascial defects were closed and the skin was resutured. Robotic partial nephrectomies were performed by daVinci Standard® surgical system until November 2013, since then by daVinci SI HD® system with near-infrared fluorescence imaging mode using a 12-mm robotic camera port, two 8-mm robotic ports and an additional 12-mm port. Renal tumours were dissected with purely off-clamp technique in cases of completely exophytic growth of tumour. The renal hilus was exposed prior to renal tumour dissection in setting of non-fully exophytic growth of tumour and on-clamp technique of partial nephrectomy was performed. In cases of unfavourable anatomical settings, the non-selective clamping of renal hilus was performed or the ICG fluorescent dye was used. Next steps of robotic partial nephrectomy (excluding renorrhaphy, which was performed with the sliding clip technique) were similar to the steps of laparoscopic partial nephrectomy, described above. In 28 cases, ICG was used for intraoperative angiography to visualise the renal tumour blood supply, administration was performed according to the manufacturer’s recommendations, as we recently described [8].

## Results

Analysis of patient characteristics showed significantly lower mean R.E.N.A.L. nephrometry score (6 vs. 7) in the group of patients who underwent laparoscopic partial nephrectomy compared to the group of patients with robotic partial nephrectomy, no significant differences were found for any other observed parameters (Table 1). We identified 27 patients (14%) with previous abdominal surgery in the upper contralateral quadrant, 63 patients (32%) with previous surgery in the upper ipsilateral quadrant and 106 patients (54%) with previous surgery in lower abdominal quadrants (Table 2). Group A included 21 patients with previous abdominal surgery in the upper contralateral quadrant. Of all 21 cases, 8 patients underwent laparoscopic partial nephrectomy and 13 patients robotic partial nephrectomy. These subgroups were similar in mean age (60 vs. 55 years), mean BMI (29.9 vs. 28.0 kg/m^2^), mean CCI (5 vs. 4) and mean average tumour diameter (26 vs. 22 mm). Significantly shorter mean surgery time (100 vs. 125 min), higher mean blood loss (238 vs. 93 mL) and lower mean preoperative glomerular filtration rate were observed in the laparoscopic subgroup. No significant differences were found in any other studied parameters. Group B contained 60 patients with previous abdominal surgery in the upper ipsilateral quadrant or in the middle line. A total of 22 patients underwent laparoscopic partial nephrectomy and 38 patients underwent robotic partial nephrectomy; the subgroups were similar in mean age (64 vs. 65 years), mean BMI (28.9 vs. 29.7 kg/m^2^), mean CCI (4 vs. 5) and mean average tumour diameter (22 vs. 26 mm). In total, 3 patients (14%) in the laparoscopic subgroup and 3 patients (8%) in robotic subgroups were discharged with significantly elevated values of creatinine compared to preoperative values. No significant differences between these subgroups were found in any of the observed parameters. Group C included 47 patients with previous abdominal surgery in lower abdominal quadrants; 11 patients underwent laparoscopic partial nephrectomy and 36 patients underwent robotic partial nephrectomy. Subgroups were similar in mean age (55 vs. 59 years), mean BMI (26.9 vs. 27.5 kg/m^2^), mean CCI (3 vs. 4) and mean average tumour diameter (34 vs. 32 mm). Significantly, shorter mean surgery time (107 vs. 130 min) and longer average length of stay in hospital (9 vs. 7 days) were observed in the laparoscopic subgroup. No significant differences were found in any of the other parameters. The type of partial nephrectomy (robotic or laparoscopic) did not significantly influence the frequency of intraoperative or postoperative complications in any of our patient groups (Table 3).

**Table 1:** Patient characteristics and tumour features

Variable	Overall (*n* = 128)	LPN (*n* = 41)	RPN (*n* = 87)	*p* value
Age (years)				
Mean, SD	61 SD12	61 SD10	61 SD12	*0.968*
Median (range)	63 (34–88)	63 (35–78)	64 (34–88)	
BMI (kg/m^2^)				
Mean, SD	28.6 SD4.9	28.6 SD4.7	28.6 SD5.0	0.999
Median (range)	27.8 (19.4–48.9)	28.1 (19.4–40.0)	27.5 (19.7–48.9)	
CCI				
Mean, SD	4 SD2	4 SD1	5 SD2	*0.268*
Median (range)	4 (2–10)	4 (2–8)	4 (2-10)	
Laterality (*n*, %)				
Left	62 (48)	19 (46)	43 (49)	*0.747*
Right	66 (51)	22 (54)	44 (51)	*0.747*
CT tumour diameter (mm)				
Mean, SD	27 SD12.6	26 SD12.6	28 SD12.6	*0.326*
Median (range)	24 (8–70)	22 (8–56)	25 (8–70)	
R.E.N.A.L. score (*n*)				
Mean, SD	6 SD1.6	6 SD1.5	7 SD1.5	***0.008***
Median (range)	7 (4–10)	6 (4–9)	7 (4–10)	

*BMI* body mass index; *CCI* Charlson comorbidity index; *CT* computed tomography; *LPN* laparoscopic partial nephrectomy; *R.E.N.A.L.* nephrometry score system; *RPN* robotic partial nephrectomy. Data presented as mean and standard deviation (SD), numbers, with percentages in parentheses, or median and range, significance *p* < 0.05 in bold

**Table 2:** List of 196 previous abdominal surgeries in 128 patients

**Previous surgical procedure**	**196 (100%)**
***Abdominal surgery in upper contralateral quadrant***	**27 (14%)**
Cholecystectomy (for left-side partial nephrectomy)	13
Contralateral partial nephrectomy	8
Contralateral nephrectomy	6
***Abdominal surgery in upper ipsilateral quadrant/in middle line***	**63 (32%)**
Cholecystectomy (for right-side partial nephrectomy)	26
Hemicolectomy	8
Ipsilateral previous partial nephrectomy	8
Umbilical hernia repair	5
Explorative laparotomy	5
Diaphragm repair	2
Gastric bandaging	2
Miles’ procedure	2
Trauma laparotomy	2
Abdominal wall reconstruction	2
Hemihepatectomy	1
***Abdominal surgery in lower abdominal quadrants***	**106 (54%)**
Appendectomy	47
Gynaecologic pelvic surgery	29
Transperitoneal inguinal hernia repair	16
Radical prostatectomy	12
Urinary bladder reconstruction	2

**Table 3:** Intraoperative and postoperative outcomes of partial nephrectomies in patients with previous abdominal surgery

Variable	Group A, *n* = 21			Group B, *n* = 60			Group C, *n* = 47		
	LPN	RPN	*p value*	LPN	RPN	*p value*	LPN	RPN	*p value*
Patients (*n*, %)	8 (38)	13 (62)		22 (37)	38 (63)		11 (23)	36 (77)	
Surgery time (min)									
Mean, SD	100 SD15	125 SD28	***0.036***	110 SD31	121 SD41	*0.318*	107 SD28	130 SD27	***0.022***
Median (range)	100 (75–120)	130 (75–165)		106 (55–180)	118 (60–235)		112 (45–145)	120 (70–185)	
Warm ischemia time (min)									
Mean, SD	13 SD2	14 SD4	*0.503*	15 SD5	14 SD5	*0.597*	18 SD3	16 SD4	*0.327*
Median (range)	13 (11–15)	17 (9–17)		16 (7–20)	12 (7–27)		18 (15–20)	15 (10–24)	
Estimated blood loss (mL)									
Mean, SD	238 SD109	93 SD44	***0.001***	238 SD184	220 SD356	*0.827*	166 SD94	182 SD272	*0.846*
Median (range)	250 (50–400)	100 (50–200)		200 (50–800)	100 (50–1500)		150 (25–300)	100 (0–1500)	
Hospital stay (days)									
Mean, SD	9 SD3	7 SD3	*0.100*	8 SD2	7 SD2	*0.261*	9 SD3	7 SD1	***0.002***
Median (range)	9 (7–16)	7 (5–16)		8 (4–11)	7 (4–16)		8 (5–15)	7 (5–10)	
Positive margins (*n*, %)	0 (0)	1 (8)	*0.447*	1 (5)	1 (3)	*0.704*	0 (0)	5 (14)	*0.214*
Intraop complication (*n*, %)									
Low-grade (CD I–II)	0 (0)	0 (0)	*–*	0 (0)	1 (3)	*0.446*	0 (0)	1 (3)	*0.587*
High-grade (CD III–V)	0 (0)	0 (0)	*–*	1 (5)	2 (5)	*0.895*	0 (0)	3 (8)	*0.341*
Postop complications (n, %)									
Low-grade (CD I–II)	1 (13)	1 (8)	*0.727*	4 (18)	6 (16)	*0.838*	3 (27)	3 (8)	*0.724*
High-grade (CD III–V)	0 (0)	0 (0)	*–*	2 (9)	1 (3)	*0.285*	0 (0)	1 (3)	*0.587*
Preop creatinine (μmol/L)									
Mean, SD	101 SD35	84 SD22	*0.328*	90 SD28	97 SD72	*0.738*	67 SD17	81 SD18	*0.164*
Median (range)	89 (74–140)	75 (65–130)		88 (39–152)	84 (49–452)		62 (51–91)	80 (50–131)	
Postop creatinine (μmol/L)									
Mean, SD	104 SD63	97 SD50	*0.762*	85 SD19	93 SD64	*0.596*	71 SD18	76 SD17	*0.470*
Median (range)	89 (58–255)	86 (49–243)		83 (55–122)	74 (45–439)		75 (42–101)	74 (45–121)	

Group A = main prior abdominal surgery in upper contralateral quadrant; group B = main prior abdominal surgery in upper ipsilateral quadrant or in the middle line; group C = main prior abdominal surgery in lower abdominal quadrants

*CD* Clavien-Dindo; *LPN* laparoscopic partial nephrectomy; *RPN* robotic partial nephrectomy

Data presented as mean and standard deviation (SD), numbers, with percentages in parentheses, or median and range, significance *p* < 0.05 in bold

Intraoperative and postoperative complications were evaluated separately for each studied group according to Clavien-Dindo classification of surgical complications. Grades I and II were stratified as low-grade complications; grades III, IV and V were stratified as high-grade complications. All complications were divided into intraoperative complications and early postoperative complications, which occurred within 30 days, and they were evaluated for each studied group separately. Intraoperative low-grade complications were observed in 2 cases (1.6 %) and were defined as excessive bleeding of the tumour bed with blood transfusion - 1 case in group B and 1 case in group C. Intraoperative high-grade complications were recorded in a total of 6 cases (4.7%), and included vessel injury (3 cases, 2.3%), intestine injury (2 cases, 1.6%) and diaphragm injury (1 case, 0.8%), all with need of suture. A total of 3 cases were observed in group B and 3 cases in group C. Postoperative low-grade complications were identified in 18 cases (14.1%), and included urinary tract infections with the necessity of the antimicrobial therapy (8 cases, 6.3%), perirenal hematoma with the need for blood transfusion without surgical intervention (5 cases, 3.9%), paralytic ileus managed by drug therapy (4 cases, 3.1%) and infection in the surgical wound managed at the bedside (1 case, 0.8%). In summary, 2 cases were recorded in group A, 10 cases in group B and 6 cases in group C. Postoperative high-grade complications were found in 4 cases (3.1%): a renal artery aneurysm with the need for radiological endovascular embolization (1 case, 0.8%), urinoma from the insufficient suture of the renal calyx with the necessity for urine drainage (1 case, 0.8%), urine retention with the need for surgical intervention (1 case, 0.8%) and renal and respiratory failure with the need for oxygen therapy and intensive care unit treatment (1 case, 0.8%) - 3 cases were found in group B and 1 case was observed in group C. After statistical analysis, we did not find any significant differences of frequency in any category of complications between the studied groups. The location of previous abdominal surgery in the upper contralateral quadrant, the upper ipsilateral quadrant (or in the middle line) or the lower abdominal quadrants did not significantly influence the rate of intraoperative or postoperative complications.

We analysed characteristics and results of 29 patients with a history of previous surgery on ipsilateral/contralateral kidney and 99 patients with previous non-renal surgery who underwent transperitoneal partial nephrectomy. Significant differences were observed in patient sex stratification, mean CCI (5 vs. 4), longer mean surgery time (132 vs. 116 min), higher intraoperative low-grade complications (2 vs. 0), higher mean postoperative creatinine (113 vs. 78) and lower mean postoperative glomerular filtration rate in group of patients with prior renal surgery. No significant differences were found in any of the other observed parameters (Table 4).

**Table 4:** Comparison of characteristics and intraoperative and postoperative outcomes in patients with a history of previous ipsi-/contralateral kidney surgery and patients with a history of previous non-renal surgery (*n* = 128)

Variable	Previous renal surgery (*n* = 29)	Previous non-renal surgery (*n* = 99)	*p value*
BMI (kg/m^2^)			
Mean, SD	29.2 SD5.1	28.4 SD4.9	*0.443*
Median (range)	27.8 (19.7–41.9)	27.7 (19.4–48.9)	
CT tumour diameter (mm)			
Mean, SD	24 SD12	28 SD13	*0.087*
Median (range)	22 (10–70)	25 (8–62)	
R.E.N.A.L. score (*n*, %)			
Mean, SD	6 SD1.3	7 SD1.6	*0.335*
Median (range)	6 (4–8)	7 (4–10)	
Surgery time (min)			
Mean, SD	132 SD44	116 SD29	***0.032***
Median (range)	130 (55–235)	116 (45–185)	
Warm ischemia time (min)			
Mean, SD	17 SD6	15 SD4	*0.138*
Median (range)	17 (7–27)	15 (7–24)	
Estimated blood loss (mL)			
Mean, SD	223 SD389	191 SD212	*0.575*
Median (range)	50 (50–1500)	100 (50–1500)	
Hospital stay (days)			
Mean, SD	8 SD3	8 SD2	*0.860*
Median (range)	7 (4–16)	7 (4–16)	
Positive margins (*n*, %)	1 (3)	7 (7)	*0.482*
Intraop complications (*n*, %)			
Low-grade (CD I–II)	2 (7)	0 (0)	***0.008***
High-grade (CD III–V)	0 (0)	6 (6)	*0.177*
Postop complications (*n*, %)			
Low-grade (CD I–II)	2 (7)	16 (16)	*0.210*
High-grade (CD III–V)	1 (3)	3 (3)	*0.910*
Preop creatinine (μmol/L)			
Mean, SD	103 SD78	82 SD21	*0.076*
Median (range)	90 (51–452)	80 (39–152)	
Postop creatinine (μmol/L)			
Mean, SD	113 SD81	78 SD20	***0.001***
Median (range)	91 (42–439)	76 (45–149)	

*BMI* body mass index; *CD* Clavien-Dindo; *CT* computed tomography; *R.E.N.A.L.* nephrometry score system. Data presented as mean and standard deviation (SD), numbers, with percentages in parentheses, or median and range, significance *p* < 0.05 in bold

We separately evaluated outcomes and complications of 28 patients with previous abdominal surgery who underwent robotic partial nephrectomy enhanced by ICG, and compared them to outcomes of 59 patients who underwent robotic partial nephrectomy without ICG imaging. These subgroups were similar in mean age (58 vs. 62 years), mean BMI (29.7 vs. 28.0 kg/m^2^), mean CCI (4 vs. 5), and in mean R.E.N.A.L. score (7 vs. 6). Significant differences were observed in higher mean CT tumour diameter (32 vs. 26 mm) and in higher intraoperative low-grade complications (2 vs. 0) in group of patients with use of ICG. No significant differences were found in all other observed parameters. Of 28 cases, the application of ICG helped to track a specific tumour-supplying artery in 19 cases where superselective clamping was performed, while 7 partial nephrectomies were completed with non-selective clamping of the renal artery. Our study did not find better intraoperative outcomes or a lower frequency of complications in cases where partial nephrectomy was enhanced by ICG imaging in the group of patients with previous abdominal surgery (Table 5).

**Table 5:** Comparison of patient characteristics, intraoperative and postoperative outcomes of robotic partial nephrectomy with ICG-enhanced robotic partial nephrectomy in setting of previous abdominal surgery (*n* = 87)

Variable	RPN with ICG (*n* = 28)	RPN without ICG (*n* = 59)	*p value*
BMI (kg/m^2^)			
Mean, SD	29.7 SD5.6	28.0 SD4.7	*0.138*
Median (range)	28.3 (21.8–48.9)	27.2 (19.7–41.9)	
CT tumour diameter (mm)			
Mean, SD	32 SD13	26 SD12	***0.030***
Median (range)	28 (13–62)	23 (8–70)	
R.E.N.A.L. score (*n*, %)			
Mean, SD	7 SD1.3	6 SD1.6	*0.121*
Median (range)	7 (4–10)	6 (4–10)	
Surgery time (min)			
Mean, SD	132 SD32	121 SD35	*0.189*
Median (range)	123 (85–235)	120 (60–230)	
Warm ischemia time (min)			
Mean, SD	15 SD5	14 SD4	*0.483*
Median (range)	15 (7–27)	15 (7–23)	
Estimated blood loss (mL)			
Mean, SD	212 SD382	177 SD250	*0.620*
Median (range)	100 (0–1500)	100 (50–1500)	
Hospital stay (days)			
Mean, SD	7 SD1	7 SD2	*0.587*
Median (range)	7 (5–10)	7 (4–16)	
Positive margins (n, %)	1 (4)	6 (10)	*0.296*
Intraop complications (*n*, %)			
Low-grade (CD I–II)	2 (7)	0 (0)	***0.038***
High-grade (CD III–V)	1 (4)	4 (7)	*0.553*
Postop complications (n, %)			
Low-grade (CD I–II)	5 (18)	5 (8)	*0.204*
High-grade (CD III–V)	0 (0)	2 (3)	*0.330*
Preop creatinine (μmol/L)			
Mean, SD	80 SD15	94 SD63	*0.369*
Median (range)	79 (55–120)	82 (49–452)	
Postop creatinine (μmol/L)			
Mean, SD	80 SD21	89 SD56	*0.427*
Median (range)	74 (52–123)	76 (45–439)	

*BMI* body mass index; *CD* Clavien-Dindo; *CT* computed tomography; *ICG* Indocyanine green; *R.E.N.A.L.* nephrometry score system; *RPN* robotic partial nephrectomy. Data presented as mean and standard deviation (SD), numbers, with percentages in parentheses, or median and range, significance *p* < 0.05 in bold

## Discussion

Minimally invasive partial nephrectomy is considered to be a standard method of therapy of localised renal cancer, with superior long-term postoperative outcomes compared to radical nephrectomy. Laparoscopic and robotic partial nephrectomy offer more favourable intraoperative outcomes and better postoperative outcome compared to open techniques. Moreover, robotic surgical systems have enabled surgeons to overcome the technical limits of laparoscopy [9, 10].

**Fig. 2:**
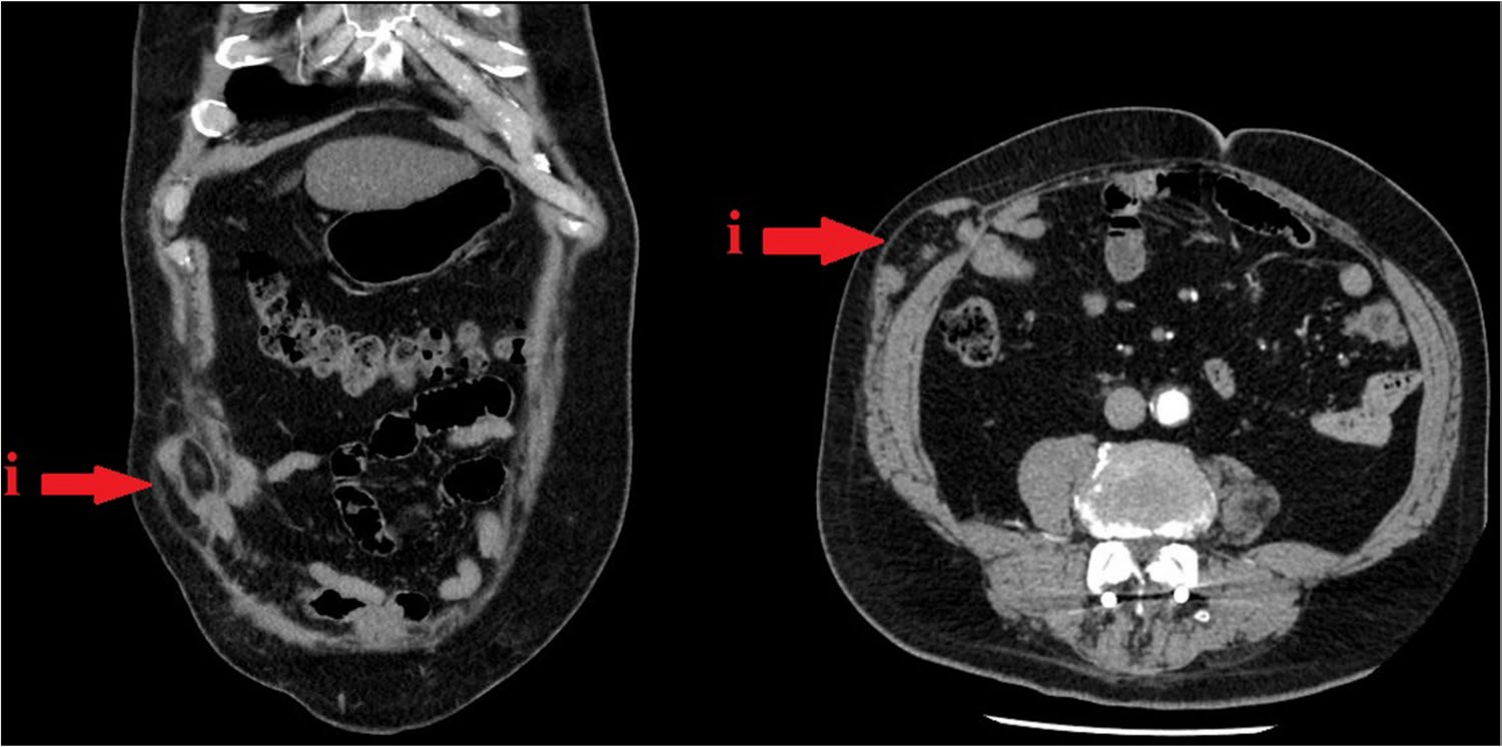
Massive incisional hernia (i) through abdominal wall on CT scan (coronary and axial projection) of patient with personal history of previous abdominal surgery - open cholecystectomy (picture from author‘s personal collection)

Peritoneal adhesions after abdominal and pelvic surgeries remain extremely common. The formation of postsurgical adhesions has been reported to occur in up to 90% of patients with a history of previous abdominal surgery. There are several aspects that contribute to these adhesions: location, type and extension of previous surgery, presence of suture material, persistence of haematoma, preoperative/postoperative infections and influence of electrocautery [11]. Other potential causes of adhesions, such as radiation therapy and inflammatory disorders, were not considered in this study. Peritoneal adhesions, caused by previous surgery can be accompanied by incisional hernias, which can also complicate or worsen the results of the subsequent surgery (Fig. 2).

**Fig. 3:**
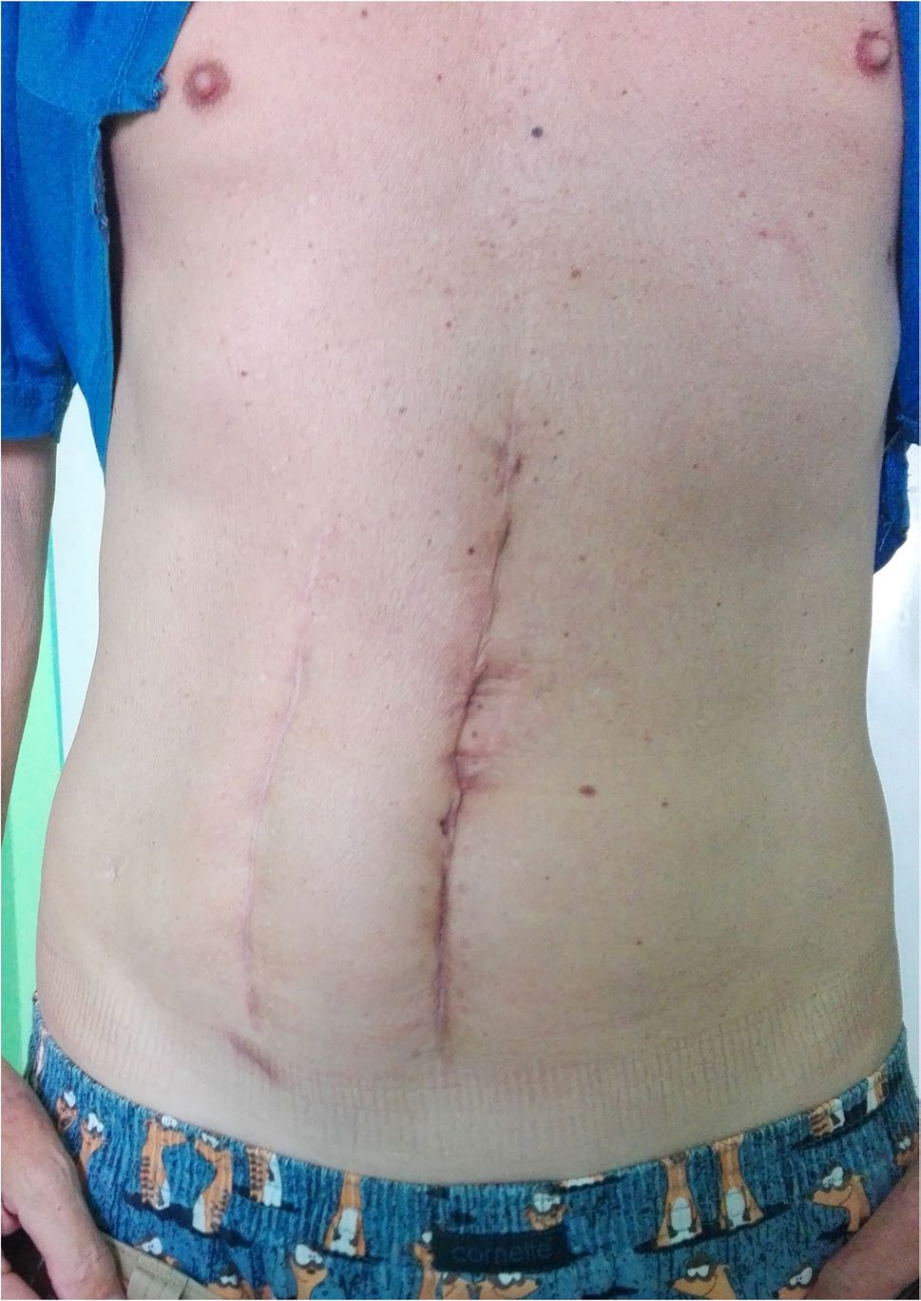
Patient with two abdominal scars after two hemicolectomies for a colorectal carcinoma and its recurrence 12 and 3 months before the robotic partial nephrectomy

Previous groups have reported the effects of previous abdominal surgery on transperitoneal laparoscopic urologic procedures with variable results [2, 12, 13]. Several researchers have published the impact of previous surgeries on transperitoneal robotic surgery [1, 3, 14]. While some authors consider patients with a previous inguinal hernia surgery as a part of group with prior abdominal surgery in common [14], other authors published outcomes of 321 patients who underwent transperitoneal robotic partial nephrectomy and they did not include inguinal hernia surgery in their group of previous abdominal surgeries [1]. In our study, we considered intraperitoneal inguinal hernia repair and umbilical hernia repairs as a part of group of previous abdominal surgeries, while extraperitoneal inguinal hernia repairs were excluded. Entry techniques in laparoscopic surgery via a Veress needle can be associated with increased rates of failed entry and visceral injury. A rate of access complications of 4% for patients undergoing renal/adrenal laparoscopic procedures via the transperitoneal approach is described in literature [15]. We did not record any complications creating a pneumoperitoneum via a Veress needle in our group of patients and an open technique was used minimally. Location of entry to peritoneal cavity was modified in cases of large abdominal scars (Fig. 3) with the aim of avoiding the access through the scarred skin.

Several groups have reported the impacts of previous abdominal surgery on different urologic robotic surgeries. One study published outcomes of robotic radical prostatectomies in patients with a history of previous abdominal surgery and showed no increased risk of complications [16]. In contrast, another study described results from 18 patients undergoing robot-assisted radical cystectomy, who had a higher risk for postoperative complications [17]. The impacts of previous abdominal surgery on transperitoneal laparoscopic procedures have also been evaluated [2, 12, 13, 15]. Some authors have described higher complication rates in patients with prior abdominal surgery [13], while others have shown no increase in complications [2, 15]. We identified one patient who underwent a retroperitoneal approach for robotic partial nephrectomy in our centre with favourable outcomes and no intraoperative or postoperative complications. We did not include this patient into our study. An extensive meta-analysis suggesting that retroperitoneal robotic partial nephrectomy appears to be equally safe and efficacious in terms of complications, conversion and oncologic control compared with transperitoneal robotic partial nephrectomy [18].

Urologic surgeons in our centre only use ICG during robotic partial nephrectomy. To facilitate this procedure, ICG can be used in three different ways. The first, which is demonstrated in our study, uses ICG to visualise of the vascular supply of the kidney. It enables the surgeon to recognise the branches of the main renal artery and perform a targeted closure of the blood flow to the relevant renal segment (selective clamping), or perform a targeted closure of the artery that supplies the tumour (superselective clamping) [19]. The second possibility takes advantage of the ability of ICG to form a chemical bond with the protein bilitranslocase (BLT), which is located in the proximal and distal renal tubules and serves as an intracellular transporter of the ICG molecule. A healthy renal parenchyma is capable of accumulating ICG molecules intracellularly and emits detectable fluorescent light. Renal tumour cells do not express BTL, ICG molecules are rapidly washed from the tumourous tissue and it does not emit fluorescent light. This imaging technique requires the administration of higher doses of ICG [5, 19]. The third method is usage of ICG as lipiodol-ICG mixture, administered intravenously before surgery and serving as a preoperative angiography agent. This angiographic mixture selectively reaches arteries of the tertiary order and enables the superselective labelling of tumour tissue. Lipiodol in the mixture counteracts the rapid reduction of ICG from tumourous tissue. The resection itself takes place without the restriction or interruption of arterial blood to the kidney [20].

## Conclusion

The location of previous abdominal surgeries did not significantly influence the rate of intraoperative or postoperative complications of subsequent partial nephrectomy in our study. The type of partial nephrectomy (robotic or laparoscopic) affected surgery time, blood loss and the length of hospital stay, but did not significantly influence the frequency of complications. Extended surgery time and higher rate of intraoperative low-grade complications during partial nephrectomy were observed in the group of patients with previous renal surgery compared to the group of with previous non-renal surgery. Our study did not find more favourable intraoperative outcomes or a lower frequency of complications in cases where indocyanine green-enhanced partial nephrectomy was performed.

## Data Availability

The dataset gathered during the present study are available from the corresponding author on reasonable request.
